# Recovery from Mild Traumatic Brain Injury in the Nonathletic Population: A Systematic Review

**DOI:** 10.1089/neur.2025.0006

**Published:** 2025-04-24

**Authors:** Véronique Déry, Gabrielle Lafond, Rosemarie Picard, Pierre Langevin

**Affiliations:** ^1^ School of Rehabilitation Science, Faculty of Medicine, Université Laval, Quebec City, Canada.; ^2^ Clinique PCN Physiothérapie, Quebec City, Canada.; ^3^ Centre Intégré Universitaire de Santé et de Services Sociaux de la Capitale Nationale, Québec, Canada.; ^4^ Centre interdisciplinaire de recherche en réadaptation et intégration sociale, Québec City, Canada.; ^5^ Clinique Cortex, Quebec City, Canada.

**Keywords:** mild traumatic brain injury or concussion, prognosis, recovery

## Abstract

The objective of this study was to document the resolution rate of mild Traumatic Brain Injury (mTBI) symptoms at various time points in a nonathletic adult population and identify prognostic factors influencing recovery. Sixteen prospective cohort studies were included, focusing on participants aged 18–65 with acute mTBI, followed for a minimum of 1 month. The recovery criterion was the resolution of symptoms not attributable to pre-existing conditions. Risk of bias was assessed using the Quality in Prognostic Studies tool, with most studies rated as moderate risk, highlighting variability in methodological rigor. Symptom resolution was reported in 49.0% to 69.5% of patients at 1 month, 40.8% to 84.4% at 3 months, 38.3% to 72.2% at 6 months, and 58.1 to 68.3% at 12 months. These findings emphasize the first 6 months as a critical period for evaluating the risk of symptom chronicity. The most commonly reported prognostic factors was baseline symptom severity, including higher intensity of symptoms such as headaches, nausea, and dizziness, as well as elevated scores on validated symptom scales. Psychiatric history, such as pre-existing anxiety or depression, was also a significant predictor of prolonged symptoms. Biomarkers, including NSE and S-100B levels, and reduced blood-derived neurotrophic factors, were associated with poorer recovery at 6 months. Demographic factors, including age, gender, and education level, showed mixed results. While some studies associated female gender, older age, and lower education with poorer recovery, others found no significant correlations. These discrepancies highlight the complexity of mTBI prognosis. Overall, more than half of patients recover within 6 months, but persistent symptoms can have a profound impact on quality of life and functional status. Identifying patients at higher risk of prolonged recovery is crucial for targeted management strategies, emphasizing the importance of individualized, evidence-based care in mTBI populations.

## Introduction

The World Health Organization defines mild traumatic brain injury (mTBI) as an acute brain injury resulting from a transfer of energy from an external source to the skull and underlying structures.^1^ In Canada, approximately 100–300/100,000 cases of mTBI are reported each year.^2^ Following a direct or indirect blow to the head, mTBI diagnosis is considered when a score between 13 and 15 on the Glasgow Coma Scale^3^ is recorded with the presence of one or more of the following symptoms: (1) loss of consciousness of <30 min; (2) post-traumatic amnesia of <24 h; (3) an altered mental state at the time of the accident, confusion, disorientation, etc.; and (4) transient neurological deficits.^1^ The diagnosis criteria have been recently revised and now also include the presence of two acute symptoms (e.g., headache, dizziness, and nausea) combined with one acute clinical sign (e.g., vestibulo-ocular impairment and balance impairment).^4^

Full recovery from symptoms following mTBI usually takes an average of 10 days to 3 months.^5^ However, some factors can negatively influence recovery, i.e., persistence of symptoms beyond 3 months. Individual factors such as age, gender, or neuropsychological history have an impact on recovery, but the number, severity, and duration of symptoms present initially and a few days after trauma are the most important predictors of recovery.^5^ Persistent symptoms can have a negative impact on the daily functioning of individuals and have significant functional consequences, thus making it an important issue in terms of public health.^6^ It is therefore interesting to identify factors that can negatively influence the prognosis in order to help clinicians in their management strategies.

A recent overview of systematic reviews^6^ included 12 systematic reviews on prognosis of recovery from mTBI and mentioned that normal resolution of symptoms occurs within 3 months, with symptoms transitioning to the chronic state beyond this period defined as prolonged recovery from mTBI. Financial compensation awarded to individuals following mTBI, absenteeism from work due to the incident, and post-traumatic symptoms of nausea or memory impairment were associated with slower recovery or persistence of symptoms. Studies from other authors suggest that the gender of the individual is a mixed factor; it was not a prognostic factor influencing post-traumatic recovery according to some,^7^ but others^8^ demonstrated that age and female gender are vulnerability factors to the persistence of postconcussion symptoms (PCS).

There is also variability in conclusions about the time required to recover from an mTBI. A systematic review by Caroll et al. reported that the recovery time for PCS is between 3 and 12 months for the adult population with age and gender as important prognostic factors,^2^ whereas another study by Machamer et al. claims that most adults recover within 3 months.^9^ There is also variability in the proportion of people who remain symptomatic over time. Indeed, the same study by Machamer et al. determined that 78% of participants in the mTBI cohort reported at least one symptom that was new or worse at 3 months than before the trauma.^9^ Nevertheless, according to a study by Skandsen et al., only 20.8% reported having postconcussion syndrome or at least three symptoms rated as moderate (score of ≥3 or a total of ≥13 on the *British Columbia Postconcussion Symptom Inventory* questionnaire).^10^ The many differences between the definition of recovery time and prognostic factors create an issue in the effective management of people with mTBI for clinicians.

The *Amsterdam International Consensus Statement on Concussion in Sports 2023* is a universally renowned reference for sport-related concussion.^11^ It defined prolonged recovery as persistence of symptoms over a month or more and describes that most athletes of all ages (93%) have a full recovery at 1 month.^12^ Recovery time is within 1 month for children, adolescents, and adults, with an average of 19.8 days. There are factors that tend to lead to poorer recoveries, such as histories of migraines and anxiety, a worsening condition, the presence of psychological stress, or social factors.^12^ The topic of prognosis of mTBI in athletes is therefore well known and widely described in the literature. However, it is possible that nonathlete population sustaining an mTBI recovers differently than athlete population because they sustained different type of injuries (e.g., intimate violence, and motor vehicle accident) or because they are more likely to have comorbidities. The access to care could also be different in athlete versus nonathlete population.

Considering the high variability previously established and the fact that the prognosis for the nonathletic population affected by mTBI is significantly less well documented than that for athletes, it is necessary to clarify the prognosis of the nonathletic population. Thus, the first objective of this systematic review was to determine the prevalence of symptom resolution in adults who have undergone mTBI in a nonsport setting, at different time points. The assumption regarding the recovery of these individuals is that a majority will recover at 3 months. The second objective was to explore the factors that influence recovery of symptoms.

## Methodology

A literature search was conducted for relevant articles in the Embase, OVID Medline, and CINHAL databases. The keywords used are presented in Supplementary Table S1. Articles published before May 31^st^, 2024, were considered. From the identified articles, the titles and abstracts were first reviewed by the four authors to determine their relevance to the research question. After this initial screening, the full texts of the remaining articles were evaluated, and the decision to include the article or not was made according to the inclusion and exclusion criteria. Covidence software was used for the screening and selection phases. All authors participated to the screening phase with a moderate level of agreement (Cohen’s Kappa = 0.41–0.48).

Eligible articles met the following inclusion criteria: (1) the study population was adults between 18 and 65 years of age, with Traumatic Brain Injury (TBI) diagnosis, of which at least 95% were mTBI; (2) prospective cohort studies with follow-up of at least 1 month; (3) more than 50% of participants had undergone their mTBI in a nonsporting setting and/or should not be considered athletes as the most recent consensus statement on sport-related concussion included articles with a population of more than 50% of concussion sustained in sports,^13^ it was the intent to retrieve different sources articles; (4) the initial assessment was completed within 2 weeks of the mTBI; and (5) solely PCS were used as a measure of recovery. Note that for studies with some moderate or severe TBI, data were analyzed with mTBI participants only when it was possible. Similarly, articles with a proportion of patients under 18 years of age or over 65 years of age were analyzed considering only patients in the targeted adult age group (18–65 years old). Articles were excluded if they met the following criteria:^1^ patients with associated conditions such as other traumatic pathologies or other neurological disorders^2^; studies with other types of designs (randomized clinical trials and retrospective studies)^3^; and studies on military populations.

In data extraction, the proportion of patients considered recovered during the various predetermined follow-ups and the prognostic factors were collected. Other collected characteristics included study location (country); year of publication; sample size; average age; proportion of male or female participants; mean time from mTBI to intake; possible prognostic factors measured at baseline; number and timing of follow-ups; and the authors’ definition of “recovery” and symptom-related outcome measures at each follow-up (Table 1). Given the significant variability in the definitions of recovery from TBI reported in the articles, it was necessary to establish the definition of recovery used in this systematic review. Therefore, consistent with the systematic review published by Putukian et al.,^12^ and considering that this study is primarily interested in symptom resolution, the recovery criterion was defined as the time to the absence of symptoms produced by the current mTBI that cannot be attributed to a pre-existing injury. This definition aligns with the definition of persistent PCS presented by Lagacé-Legendre et al.,^30^ which defines it as “the presence of any symptom that cannot be attributed to a pre-existing condition and that appeared within hours of a head injury, and is still present every day 3 months after the injury, and impacts at least one area of a person’s life.”

**Table 1.** Characteristics of Included Studies

First author, year, country	Article title	Participants (*N* at baseline, mean age, sex, time since mTBI)	Time and number of participants in each follow-up	Factors measured initially	Symptom-related measures at each follow-up and definition of “recovered” in the article	Reported prognostic factors	Reported results
Bosak, N 2022 USA^14^	Brain Connectivity Predicts Chronic Pain in Acute Mild Traumatic Brain Injury	**N:** 105 mTBI **Average age:** Approximately 35.9 years (18–70 years) **Sex:** 55 M/50 F **Time:** <72 h	**3 months:** 84 mTBI **6 months:** 93 mTBI **12 months:** 105 mTBI	- Demographic Questionnaires - Medical history - Assessment of Psychological Elements Related to Pain - Psychophysical tests - EEG - Blood test for DNA and RNA - Resting-state Functional MRI (Rs-fMRI) - Average and maximum pain in the head and neck (scale 0–100)	**Pain Symptoms** Record mean and maximum pain experienced on a numeric scale of 0–100 in the head and neck (1x/month for 12 months) **Definition of “recovered” in the article:** Mild pain (<30 on the pain scale) experienced at the end-point.	**Prognostic factors for poorer recovery at 12 months:** - Initial high pain value - Lower level of education - Decreased connectivity of the sensorimotor system with periaqueductal gray matter (PAG) and nuclei accumbens (NAc) on fMRI	**Proportion of asymptomatic subjects:** **3 months:** NR **6 months:** NR **12 months:** 61/105 (58.1%)
Chen Y-C 2022 Taiwan^15^	Personalized Prediction of Postconcussive Working Memory Decline: A Feasibility Study	**N:** 70 mTBI and 48 controls **Average age:** mTBI: 37.9 ± 12.2 years Controls: 37.4 ± 12.0 years **Sex:** mTBI: 47 M/23 F Controls: 32 M/16 F **Time:** <1 week	**6 weeks (52.52 ± 6.95 days):** 34 mTBI **3 months (100.96 ± 13.56 days):** 29 mTBI **6 months (196.95 ± 14.61 days):** 28 mTBI **12 months (376.48 ± 16.52 days):** 25 mTBI	**Demographics** **Functional MRI:** - Preprocessing - WM-Task Activation and Deactivation Map **Working Memory Assessment:** - Working Memory Index (WMI) - Arithmetic ability (AMT) - Digit span score (DS) in the WAIS-IV **Neuropsychological Assessment:** - Mini-Mental State Examination (MMSE) - Wechsler Adult Intelligence Scale fourth edition (WAIS-IV) **Clinical symptoms**: - Glasgow Outcome Scale Extended (GOSE) - Pittsburgh Sleep Quality Index - Epworth Sleepiness Scale - Dizziness Handicap Inventory - Rivermead Post Concussion Symptoms Questionnaire (RPQ) - Beck Anxiety Inventory - Beck Depression Inventory	Rivermead Post Concussion Symptoms Questionnaire (RPQ) **Definition of “recovered” in the article:** Participants with an improvement in their WMI score between the final and baseline measurement.	NR	**Proportion of asymptomatic subjects:** **6 weeks:** NR **3 mois :** NR **6 mois :** NR **12 months:** NR **Results related to working memory:** - 9/24 (38%) not recovered at 3 months post-mTBI - 18/24 (75%) have a decline between 3 months and 6 months post-mTBI - 9/24 (38%) do not recover between 6 months and 1 year post-mTBI - 11/24 (46%) had a worse outcome at 1 year post-mTBI compared with baseline
Cnossen, M.C. 2017 USA^16^	Development of a Prediction Model for Post-Concussive Symptoms following Mild Traumatic Brain Injury: A TRACK-TBI Pilot Study	**N:** 277 mTBI **Median age:** 42 years (26–57 years) **Sex:** 194 M/83 F **Time:** <24 h	**6 months:** 277 mTBI	**Demographics** - Age - Sex - Years of education - Pre-trauma seizures - Pre-accident migraine - Headaches - Pre-Accident Psychiatric Problem - History of mTBI **Computed tomography (CT scan)**	**At 6 months post-accident:** - The Rivermead Post Concussion Symptoms Questionnaire (RPQ) - Brief symptom Inventory-18 Item - Post-traumatic stress disorder Checklist-Civilian Version (PTSD Checklist) **Definition of “recovered” in the article:** Absence of postconcussive syndrome (PCS) PCS: ≥ 3 symptoms present for at least 4 weeks	**Factors associated with a poorer prognosis at 6 months:** - Age - Female - Low level of education - Pre-accident migraine/headache - Pre-Accident Psychiatric Problem - History of mTBI - APT (Post-Traumatic Stress Amnesia) - Loss of consciousness	**Proportion of asymptomatic subjects: 6 months:** 130/277 (47%)
De Kruijk, J.R 2002 England^17^	Prediction of post-traumatic complaints after mild traumatic brain injury: early symptoms and biochemical markers	**N:** 107 mTBI **Median age:** 37.2 years **Sex:** 61 M/46 F **Time:** <6 h	**2 weeks:** 103 mTBI **6 months:** 79 mTBI	**Demographics** **Symptoms present in the emergency department:** headache, dizziness, nausea, vomiting, and neck pain Serous biomarkers: NSE and S-100B **Possible Confounders:** Age, Gender, and Intervention	Severity of 16 “post-traumatic complaints” (PTC), on a visual analogue scale (VAS) in 4 subcategories: cognitive, vegetative, dysthymic and physical. (At 2 weeks: also question the severity of these symptoms before the trauma) **Definition of “recovered” in the article:** VAS of all PTCs after 6 months are below the 95th percentile of all patients’ pre-trauma VAS scores	**Factors associated with higher symptom severity at 6 months:** - Headache, dizziness or nausea in the emergency department after mTBI - Elevated serous biomarkers	**Proportion of asymptomatic subjects:** **2 weeks**: NR **6 months:** 57/79 (72.2%)
Faux, S. 2008 Australia^18^	A prospective controlled study in the prevalence of post-traumatic headache following mild traumatic brain injury	**N:** 100 mTBI and 100 controls **Average age:** TBI: 33.64 years Controls: 32.15 years **Sex:** mTBI: 78M/22F Controls: 77 M/23 F **Time:** (on average) mTBI: 13.13 h Checks: 15.25 hrs	**1 month:** 82 mTBI 92 controls **Three months:** 77 mTBI 89 controls	- Demographics - Rivermead Post Concussion Symptoms Questionnaire (RPQ) - Analogous Visual Scale - Galveston Orientation and Amnesia Test (GOAT) - Modified Westmead Post Traumatic Amnesia Scale - Modified Rapid Screen of Concussion - Modified Balance Error Scoring System	Telephone response of participants with mTBI to RPQ Question 1 (Headache Question) Pain level on the visual analogue scale (headache intensity for the mTBI group) **Definition of “recovered” in the article:** No reported headache, i.e., the answer “0– not experienced at all” to question 1 of the RPQ	NR The objective of the study was to establish the prevalence of post-mTBI headaches without analyzing prognostic factors.	**Proportion of asymptomatic subjects:** **1 month:** 57/82 mTBI (69.51%) **3 months:** 65/77 mTBI (84.42%)
Heitger, M.H. 2007 New Zealand^19^	Mild head injury—a close relationship between motor function at 1 week post-injury and overall recovery at 3 and 6 months	**N**: 37 mTBI **Average age**: 29.1 years (range 15 to 56 years) **Sex:** 24 M/13 F **Time**: within a week (average 5.5 ± 3.0 days)	**3 months (90 ± 5.5 days):** NR **6 months (182 ± 15 days):** NR	**Demographics** **Engine Tests:** - Oculomotor tests (reflexive saccades, antisaccades, memory-guided sequences and self-paced saccades) - Visuomotor tests of the upper limbs **Neuropsychological tests:** - Attention - Working Memory - Episodic Memory - Speed of information processing **Health Measures:** - SF-36 Health survey - RPSQ (Rivermead Postconcussion Symptoms Questionnaire) - RHIFQ (Rivermead Head Injury Follow-up Questionnaire)	- Summary score on the RPSQ - Summary score on the RHIFQ **Definition of “recovered” in article:** NR	**Factors associated with the level of recovery and % of variance explained by the factor:** **At 3 months:** - Motor performance (combination of oculomotor and visuomotor tests): 85–87% - Health status (SF-36 scales and Rivermead questionnaires): 54–79% **At 6 months:** - Motor performance (combination of oculomotor and visuomotor tests of MS): 71–85% - Health status (SF-36 scales and Rivermead questionnaires): 68–76%	**Proportion of asymptomatic subjects:** **3 months:** NR **6 months:** NR
Korley, K.F 2016 USA^20^	Circulating Brain-Derived Neurotrophic Factor Has Diagnostic and Prognostic Value in Traumatic Brain Injury	**N:** 311 - 76 mTBI from John Hopkins Hospital (JHH) - 76 mTBI from San Francisco General Hospital (SFGH) - 159 mTBI from the TRACK-TBI pilot study - 150 controls from JHH **Average age:** JHH : 47 years (30–56) SFGH: 42 years (26–56) TRACK-TBI: 41 years (25–56) JHH Controls: 54 years (47–62) **Sex:** JHH : 47 M/29 F SFGH: 54M/22F TRACK-TBI: 114 M/45 F Controls JHH 71 M/79 F **Time:** <24 h	**6 months:** 159 TRACK-TBI subjects (94 with RPQ results)	- CT scan - Serum sample (BDNF: blood-derived neurotrophic factor) - Demographics - Glasgow Scale	Rivermead Postconcussion Questionnaire (RPQ) **Definition of “recovered” in the article:** Recovery after 6 months post-trauma: RPQ score of <3 or a GOS-E >8.	**Factors associated with the risks of incomplete recovery at 6 months:** Very low levels of BNDF in the blood at the time of mTBI	**Proportion of asymptomatic subjects:** **6 months:** 36/94 (38.3%)
Lee, M-Y. 2023 Corée^21^	Proteomic discovery of prognostic protein biomarkers for persisting problems after mild traumatic brain injury	**N:** 42 mTBI **Average age:** 50.6 years **Sex:** 23 M/19 F **Time:** 9.6 days	- <72 h: NR - 1 week: NR - 1 month: 40 mTBI - 3 months: 40 mTBI - 6 months: 34 mTBI	- Demographics - RPCQ (Rivermead Postconcussion Symptoms Questionnaire)) - GOSE (Glasgow Outcome Scale-Extended) - BDI-II (Beck Depression Inventory-II) - K-MoCA (Korean-Montreal Cognitive Assessment) - FAB (Frontal Assessment Battery) - Serum sample	At 6 months postaccident: - RPCQ - GOSE **Definition of “recovered” in article:** Patients are categorized as having a “Good Recovery” if they do not have PCS. To determine the presence of PCS, the 8 ICD-10 criteria were used using responses to 8 of the 16 RPCQ questions. If a symptom had a RPCQ score of 2 or higher, the symptom was considered present.	- Factors associated with the risk of poor recovery, in relation to the RPCQ result at 6 months: - Proteins associated with a high and low risk of poor recovery (high risk/low risk): - <72 h: 31 proteins (15/16) - 1 week: 43 proteins (31/12) - 1 month: 15 proteins (8/7) - 3 months: 26 proteins (9/17)	- Proportion of subjects with good recovery according to the RPCQ: - 1 month: 22/40 (55.0%) - 3 months: 22/40 (55.0%) - 6 months: 19/34 (55.9%)
Losoi, H. 2016 Finland^22^	Recovery from Mild Traumatic Brain Injury in Previously Healthy Adults	**N:** 75 mTBI and 40 controls **Average age:** TBI: 37 years Controls: 40 years **Sex:** mTBI: 46 M/29 F Controls: 20 M/20 F **Average time:** 48.1 h (2.0–241.0h)	**1 month:** 74 mTBI **6 months:** 71 mTBI **12 months:** 60 mTBI	- TBI data: time and mechanism of injury, alcohol intoxication, presence and duration of loss of consciousness, presence and duration of PTA. - Demographics - Neurological examination: cranial and spinal nerves, coordination, balance, pronator drift test, adiadochokinesia, and MRI - Total Severity: Injury Severity Score (ISS).	Symptom Measurements: - RPCS (Rivermead Postconcussion Symptoms Questionnaire) - BNI-FS (Barrow Neurological Institute Fatigue Scale) - ISI (Insomnia Severity Index) - RNBI (Pain Subscale of the Ruff Neurobehavioral Inventory) - PCL-C (PTSD-Checklist, Civilian Version) - BCI-II (Beck Depression Inventory-Second Edition) **Definition of “recovered” in the article:** They use a multidimensional recovery definition: Patients without moderate PCS, without cognitive difficulty, and who have returned to work are considered with favorable recovery.	**Risk Factor for a Poor Prognosis:** No factor is significant Patients with mild postconcussion symptoms at 12 months did not have a more severe initial injury than those without symptoms at 12 months, and there were no differences in age, education, or sex between these two groups.	**Proportion of subjects without postconcussion symptoms:** - At 1 month: 47/74 (63.5%) - At 6 months: 49/69 (71%) - At 12 months: 41/60 (68.3%)
Madhavan, R. 2019 USA^23^	Longitudinal Resting State Functional Connectivity Predicts Clinical Outcome in Mild Traumatic Brain Injury	**N:** 91 mTBI and 23 controls **Age:** mTBI: 23.4 years ±8.8 Controls: 25.1 years ±8.7 **Sex:** mTBI: 49M/42F Controls: 11M/12F **Time:** <72 h	**E1: <72 h:** 28 mTBI 16 controls **E2: 5–10 days:** 67 mTBI 12 controls **E3: 15–30 days:** 54 mTBI **E4: 83–103 days:** 41 mTBI	- Demographics - Head Movement During rs-fMRI - Resting state functional MRI (rs-fMRI) (fractional amplitude of low frequency fluctuations (fALFF) et seed-based connectivity (SBC)) - Three-dimensional (3D) T1-weighted SPGR BRAVO scan - Sport Concussion Assessment Tool-2 (SCAT-2) - Symptom Severity Score (SSS)	Sport Concussion Assessment Tool-2 (SCAT-2) - Symptom Severity Score (SSS) **Definition of “recovered” in the article:** Participants with an SSS score <10 are considered recovered (with no or few symptoms).	**Prognostic factors at E4 (83–103 days):** Functional connectivity of the following brain regions to the SBC (measured on fMRI): - Dorsal Attention Network (SEED) Correlated to Motor Region (ROI) - Dorsal Attention Network (seed) correlated with the Dorsal Attention Network (ROI) region - Fault mode network (seed) correlated with motor region (ROI) - Motor Region Correlated Executive Control (ROI) Network (ROI) - Motor region (seed) correlated to the fault mode network region (ROI) - Visual region (seed) correlated to the executive control (ROI) network region SBC: seed-based connectivity ROI: region of interest	**Proportion of subjects with SSS <10 to 3 months (E4):** - 65/91 mTBI (71.4%)
Rabinowitz, A.R. 2015 USA^24^	Prevalence and Predictors of Poor Recovery from Mild Traumatic Brain Injury	**N:** 87 mTBI, 75 orthopaedic controls (OC) and 44 typically developing controls (TD) **Age:** between 12 and 30 years old **Sex:** mTBI: 42M/24F OC: 46M/17F TD: 21M/17F **Time:** <96 h	**3 months:** 66 mTBI 63 OC 38 TD	**Demographics:** - Age - Sex - Socioeconomic status - Race - Mechanism of Injury - Duration of loss of consciousness - Duration of post-traumatic amnesia - Timing of return to work/school **Symptoms and Cognitive Function:** - Rivermead Post Concussion Symptoms Questionnaire - Brief Symptom Inventory (BSI) + Global Severity Index (GSI) - Brief Visual Memory Test-Revised30 immediate recall (BVMT-R Total) - Verbal Selective Reminding Test, including the number of words recalled after a delay (VRST Delay) - Verbal Selective Reminding Test, including the number of words continuously recalled (VSRT CLTR) - Symbol-Digit Modalities Test oral (SDMT-O) - Symbol-Digit Modalities Test written (SDMT-W)	Rivermead Post Concussion Symptoms Questionnaire Brief Symptom Inventory (BSI) + Global Severity Index (GSI) **Definition of “recovered” in the article:** Symptom-related: not having symptom questionnaire results higher than the 90th percentile of controls with an orthopedic injury (OC).	**Prognostic factors for unfavorable symptomatic course 3 months post-mTBI:** Be older (young adults, 18–30 years old in this study) Being a woman Have acute symptoms in the first few days post-mTBI (<96 h)	**Proportion of asymptomatic subjects:** **3 months:** 32/66 (48.5%)
Richey, L.N. 2020 USA^25^	Age differences in outcome after mild traumatic brain injury: results from the HeadSMART study	**N:** <65 years: 359 mTBI > 65 years: 88 mTBI (group not analyzed here) **Average Age:** <65 years: 37.2 years **Sex:** <65 years old: 216 M/143 F **Time:** <24 h	**1 month:** <65 years: 259 mTBI **Three months:** <65 years: 239 mTBI **6 months:** <65 years: 221 mTBI	- Demographics - Clinical Injury Information - TBI severity (head Abbreviated Injury Scale [head AIS) - Glasgow Scale - Neurological sequelae (CT scan) - Mechanism of Injury - Intoxication at the time of the incident	- Postconcussion Symptoms (Rivermead Postconcussion Questionnaire) - Depressive symptoms (Patient Health Questionnaire–9) **Definition of “recovered” in the article:** People without postconcussion symptoms. PCS are defined here as the presence of mild, moderate, and/or severe problems in at least 2 of the following RPQ domains: (1) headache, dizziness, general malaise, excessive fatigue, or noise intolerance; (2) irritability, emotional lability, depression or anxiety; (3) subjective complaints of difficulty concentrating or remembering; (4) insomnia; (5) reduced alcohol tolerance; (6) Concern about these symptoms and fear of permanent brain damage.	**Risk Factor for a Poor Prognosis:** Age was NOT identified as a significant factor. The 65-year-group had equivalent results in terms of functional recovery and post-concussion and depressive symptoms as the <65-year-old group, at all three time points.	**Proportion of asymptomatic subjects <65 years of age:** **1 month:** 127/259 (49.0%) **3 months:** 119/239 (49.8%) **6 months:** 114/221 (51.6%)
Schneider, A.L.C. 2022 USA^26^	Cognitive Outcome 1 Year After Mild Traumatic Brain Injury: Results from the TRACK-TBI Study	**N:** 656 mTBI and 156 controls **Average Age:** TBI: 40 years Controls: 37 years **Sex:** mTBI: 416 M/240 F **Time:** <24 h	**2 weeks:** NR **6 months:** NR **12 months:** 656 mTBI 156 controls	**Demographics** **Factors measured in the laboratory:** - Blood glucose level - Blood Alcohol Concentration - Toxicology **Factor related to mTBI:** - Glasgow Coma Scale (GCS) - Mechanism of Injury - Loss of consciousness - Altered consciousness - Post-traumatic amnesia - Low blood pressure - Hypoxia - Level of Care (ICU, Service Unit, Discharged) - Ct - Rotterdam CT score	- Rivermead Post Concussion Symptoms Questionnaire - 18-item Brief Symptom Inventory Global Severity Index (BSI-18-GSI) **Definition of “recovered” in the article:** Participants with mTBI who do not have cognitive impairment, cognitive decline, or both 1 year post-mTBI.	No prognostic factors related to symptoms **Prognostic factors for unfavorable cognitive outcome 1 year post-mTBI:** - Be Black or other - Have a low level of education - Have a low annual family income - Not having health insurance - Hyperglycemia - Suffering from depression before mTBI - High injury severity (low SCT score)	**Proportion of asymptomatic subjects:** NR **Mean outcome of subjects with favorable cognitive evolution at 12 months** (*n* = 570): - RPQ: 13.9 ± 0.6 - BSI-18: 48.3 ± 0.5
Shetty, T. 2018 USA^27^	Clinical Findings in a Multicenter MRI Study of Mild TBI	**N:** 111 mTBT and 34 controls **Age:** 23.2 ± 9 years **Sex:** 63 M/48 F **Time:** E1 <72 h (48 individuals) E2: 5–10 days post-trauma (63 individuals)	Maximum of 4 times during 3 months **E1: <72h:** 48 mTBI **E2: 5–10 days:** 99 mTBI **E3: 15–29 days:** 83 mTBI **E4: 93–97 days:** 59 mTBI	- Age - Sex - Education level - Ethnic group - Race - Smoking status - Family and medical history (psychiatric illness, learning disability) - History of mTBI - Migraines or other headaches - Examen clinique : Composite General Symptoms Assessment (CGSA) - Neurological Assessment: Immediate Memory (5-Word Recall), Concentration (Digits Backward), and Balance (Modified BESS) - MRI	- Composite General Symptoms Assessment (CGSA) (includes the Sports Concussion Assessment Tool 2 and the Concussion graded symptoms checklist) - Symptom Severity Score (SSS) **Definition of “recovered” in the article:** Participants were considered recovered if they did not have cognitive impairment within 3 months.	**Factors of poor prognosis at 3 months post-mTBI:** - Female - ≥25 years - History of depression, anxiety, or other psychiatric disorders	**Proportion of asymptomatic subjects :** **3 months:** NR
van Veldhoven, L.M. 2011 USA^28^	Predictive ability of preinjury stressful life events and post-traumatic stress symptoms for outcomes following mild traumatic brain injury: analysis in a prospective emergency room sample	**N:** 222 mTBI **Mean age (participants with 3-month follow-up only):** 33.63 years **Sex (participants with 3-month follow-up only):** 143 M/43 F **Time:** <2 weeks	**3 months:** 186 mTBI	Demographics Measurement scales: - Center for Epidemiological Studies Depression Scale (CES-D) - Stressful Life Events Questionnaire (SLESQ) - PTSD Checklist (PCL)	- Brief Symptom Inventory (BSI): Depression and Anxiety Subscale Scores **Definition of “recovered” in the article:** NR	**Prognostic Factor for BSI Depression and Anxiety and Physical and Mental Health Scores at SF-36 at 3 Months:** - The incidence of stressful life events (assessed by the SLESQ)	**Proportion of asymptomatic subjects:** **3 months:** NR
Yue, J.K. 2021 USA^29^	Predictors of 6-month inability to return to work in previously employed subjects after mild traumatic brain injury: A TRACK- TBI pilot study	**N:** 152 mTBI **Mean age:** 40.7 ± 15.0 years **Sex:** 111 M/41 F **Time:** <24 h for CT scan, no details for other measurements	**3 months:** 152 mTBI **6 months:** 152 mTBI	- Demographics - Glasgow Coma Scale (GCS) in the emergency department - Computed tomography (CT scan) - Polytraumatized (yes or no) - Glasgow Outcome Scale-Extended (GOSE)	Acute Concussion Evaluation (ACE) (measured only at 3 months) **Definition of “recovered” in the article:** Their definition is specific to return to work, but regarding symptoms, their definition of a recovered patient would be the absence of a positive result for one of the 4 domains of ACE (1-physical symptom, 2- cognitive, 3- related to sleep and 4- related to emotions)	No prognostic factors related to symptoms **Factors predicting inability to return to work 6 months post-mTBI:** - Not being able to go back to work at 3 months - Have a positive result in any area of ACE 3 months post-mTBI - Have a positive result for all 4 domains of the ACE 3 months post-mTBI - Have a positive outcome in the emotional domain of ACE 3 months post-mTBI	**Proportion of asymptomatic subjects according to the different domains of the ACE:** **Three months:** - Physical symptom: 62/152 (40.8%) Other areas of ACE: - Sleep related: 80/152 (52.6%) - Cognitive symptom: 82/152 (53.9%) - Emotion related: 104/152 (68.4%) **6 months:** Not measured

mTBI, mild traumatic brain injury; N, number of participants; NR, not reported.

Subsequently, each article was subjected to a risk of bias assessment by at least two authors, using the Quality in Prognostic Studies (QUIPS) Risk of Bias Assessment Tool for Prognostic Factor Studies.^31^ This tool divides the assessment of risk of bias into six domains of potential bias: study participation, study attrition, measures of prognostic factors, outcome measures, study confounding factors, and statistical analysis. Each domain was assigned a low, moderate, or high risk of bias rating based on whether or not the characteristic was present in the study. Following the assessment of the six domains, a judgment was made on the overall risk of bias of the study using the same ratings.

By combining the results of each study, while also taking into consideration the determined risk of bias as well as the characteristics of each study (e.g., the study population), conclusions could be drawn to determine the most significant prognostic factors and the proportions of patients with resolved symptoms at different times of recovery following mTBI. To support this, the collected data were analyzed using the mean and standard deviation (SD) of population size of the included studies, as well as the average number of follow-ups per study, the range, and total proportion of asymptomatic patients at 1, 3, 6, and 12 months. A qualitative analysis was also performed to describe the prognostic factors reported by the different studies and considered significant.

## Results

**FIG. 1.**
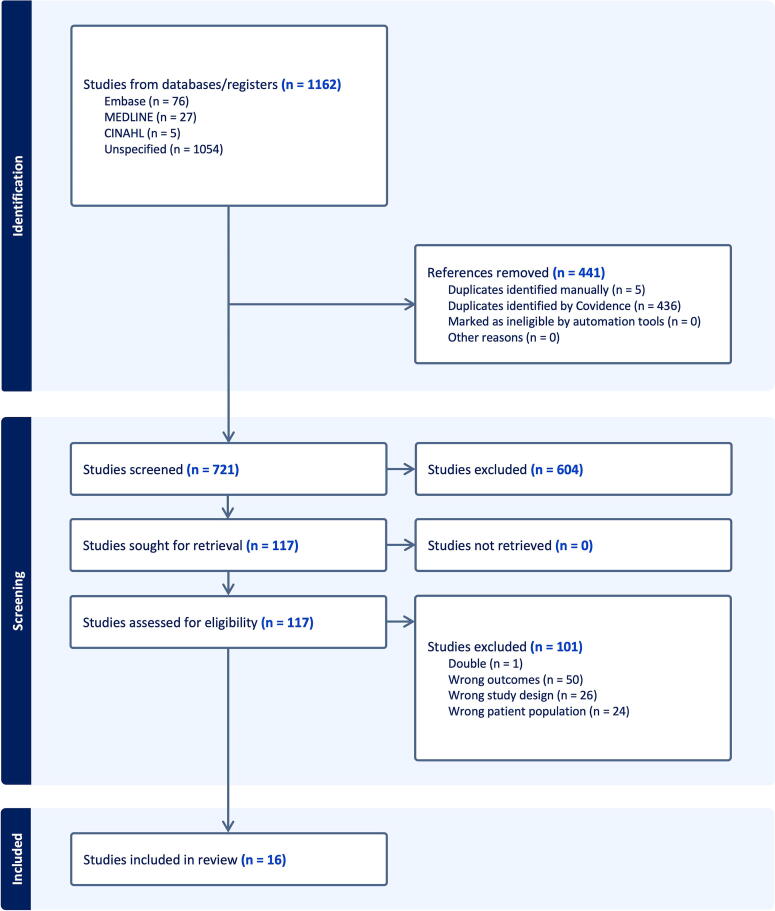
Study flow chart.

The literature search identified 1162 eligible studies (603 in Embase, 423 in Medline, and 136 in Cinhal; Supplementary Table S1). After removal of duplicates, 721 studies met the search criteria. Following revision of titles and abstracts, 117 articles were reviewed in full and finally, 16 articles^14–29^ were selected according to the inclusion and exclusion criteria (Fig. 1).

### Characteristics of included studies

Table 1 presents the characteristics of the 16 included studies. Half of the studies included a cohort of mTBI participants, while the other half included a comparison group. Ten studies included a population of <115 participants, five studies included between 150 and 500 participants, and only one study included more than 500 participants (Table 1). On average, 175.1 (SD = 163.1) participants with mTBI took part in the studies, demonstrating a very wide distribution of populations. Follow-up time points vary between 2 and 52 weeks. The studies performed an average of 2.4 follow-ups. Regarding symptom measurements at follow-up, the most commonly used tools were the Rivermead Postconcussion Questionnaire (RPQ; 10 studies) and the Brief Symptom Inventory (BSI; 4 studies). The Beck Depression Inventory was used in three studies, while the following tests were used by two: the Global Severity Index, a pain scale (visual analogue scale or numeric scale), the Post-Traumatic Stress Disorder Checklist, and the Symptom Severity Score (SSS) from the Sport Concussion Assessment Tool-2. Ten other tools were also used once, either independently or in combination with an aforementioned test (Table 1).

### Risk of bias assessment

The risk of bias of each study is presented in Table 2. Ten studies^14–17,19,22,24,26,27^ were classified as moderate risk, i.e., had no major bias but were of high or moderate risk in enough domains to raise doubts about the bias present in the study and the reliability of their results. Four studies^18,21,23,25^ were categorized as low risk of bias, which required for the studies to have taken steps to minimize the risk of bias in most of the domains of the QUIPS tool. In contrast, two studies^28,29^ had a “high” overall risk of bias, having either a major bias in one or more domains, or “moderate” or “high” risk in a majority of domains.

**Table 2.** Risk of Bias Assessment of Studies Included with the QUIPS Tool

Categories articles	1. Participation in the study	2. Study attrition	3. Measurement of prognostic factors	4. Measuring results	5. Confounding factors of the study	6. Statistical analysis	Overall risk of bias
Bosak et al. (2022)^14^	Moderate	High	Low	Low	High	Low	Moderate
Chen et al. (2022)^15^	Low	Low	Low	Low	Low	Moderate	Low
Cnossen et al. (2017)^16^	Low	Moderate	Low	Low	High	Low	Moderate
de Kruijk et coll. (2002)^17^	Low	Moderate	Low	Low	High	Low	Moderate
Faux et al. (2008)^18^	Low	High	N/A	Low	Moderate	High	High
Heitger et al. (2007)^19^	Moderate	High	Low	Low	High	Low	Moderate
Korley et coll. (2016)^20^	Low	High	Low	Low	High	Low	Moderate
Lee et al. (2023)^21^	Low	High	Low	Low	High	Low	Moderate
Losoi et al. (2016)^22^	Low	Moderate	Low	Low	Low	Low	Low
Madhavan et al. (2019)^23^	Moderate	High	Moderate	Low	High	Moderate	High
Rabinowitz et coll. (2015)^24^	Low	High	Low	Low	Low	Low	Moderate
Richey et al. (2020)^25^	Low	High	Low	Low	Moderate	Low	Moderate
Schneider et al. (2022)^26^	Low	Moderate	Low	Low	High	Low	Moderate
Shetty et coll. (2018)^27^	Low	High	Low	Moderate	High	Low	Moderate
van Veldhoven et coll. (2011)^28^	Low	Moderate	Low	Low	Low	Moderate	Low
Yue et al. (2021)^29^	Low	Low	Low	Low	High	Low	Low

QUIPS, Quality in Prognostic Studies.

In addition, some areas of the QUIPS tool were more frequently identified as “high risk” or “low risk.” First, most studies were found to have a low risk in the “Prognostic Factor Measurement” (14 out of 16 studies) and “Outcome Measurement” (15 out of 16 studies) domains. Indeed, the use of valid and reliable tools was widespread in the studies. In both cases, data collection was conducted in person, by telephone, or through self-administered questionnaires. In addition, clear definition of the prognostic factors or outcome measures was consistently used. In contrast, high risk was often noted for the domains “Cconfounding factors” (10 out of 16 studies) and “Study attrition” (9 out of 16 studies) as data on participants who dropped out during follow-up were often missing. Few studies described attempts to collect information from these participants or the reasons for their dropout. Only a few articles reported key characteristics of lost participants or noted that there were no significant differences between those who completed the study and those who did not. Regarding attrition, although the proportion of participants who completed the studies was sometimes satisfactory, several pieces of information on participants attrition were often missing. It was also rare to find a description of attempts to collect information from them or the reasons for the loss to follow-up.

### Proportions of recovered patients

As different studies used different outcome measures, it was not possible to pool the data across studies. The results of each study with proportion of patients recovered at 1, 3, 6, and 12 months are shown in Table 3.

**Table 3.** Proportions of Participants Who Recovered at 1, 3, 6, and 12 Months with the Overall Risk of Bias of the Study Represented with Colors

Articles	1 month		3 months		6 months		12 months	
	*n* recovered/*n* total	Proportion recovered (%)	*n* recovered/*n* total	Proportion recovered (%)	*n* recovered/*n* total	Proportion recovered (%)	*n* recovered/*n* total	Proportion of *n* recovered (%)
Bosak (2022)^14^							61/105	58.1%
Cnossen (2017)^16^					130/277	46.9%		
de Kruijk (2002)^17^					57/79	72.2%		
Faux (2008)^22^	57/82	69.5%	65/77	84.4%				
Korley (2016)^20^					36/94	38.3%		
Lee (2023)^21^	22/40	55.0%	22/40	55.0%	19/34	55.9%		
Losoi (2016)^22^	47/74	63.5%			49/69	71.0%	41/60	68.3%
Madhavan (2019)^23^			65/91	71.4%				
Rabinowitz (2015)^24^			32/66	48.5%				
Richey (2020)^25^	127/259	49.0%	119/239	49.8%	114/221	51.6%		
Yue (2021)^29^			62/152	40.8%				

Risk of bias legend: green = low; yellow = moderate; red = high.

Four studies reported a proportion of asymptomatic patients at 1 month, and proportions of asymptomatic participants ranged from 49.0% to 69.5%. Six studies reported a proportion of asymptomatic patients at 3 months, with a range of participants with full recovery between 40.8% and 84.4%. Subsequently, six studies presented prognostic data at 6 months and the proportion of recovered patients at 6 months ranged from 38.3% to 72.2%. Finally, at 12 months post-mTBI, two studies reported data to establish a proportion of recovered patients ranging between 58.1% and 68.3%.

### Prognostic factors

A wide variety of factors were reported to be predictive of symptom persistence (Table 1). The most common factor, reported by four studies, was baseline symptomatology, i.e., the number and intensity of symptoms present at baseline. However, this was expressed differently in different articles. Predictive symptoms can therefore vary; these include high pain intensity,^14^ headache, nausea, or dizziness,^17^ or high scores on symptom questionnaires such as the RPQ or BSI.^19,24^ Notably, all four studies that identified symptom severity as a prognostic factor measured it within the first week postinjury. In contrast, six studies^16,20,21,23,25,26^ did not assess symptoms at baseline, and two studies^18,29^ did not report prognostic factors. Three additional studies^15,22,28^ measured symptoms at baseline but focused on other specific prognostic factors. Only one other study^27^ assessed symptom severity within 72 h postinjury and did not find it to be a prognostic factor.

Another common factor is female gender, reported by three studies which identified that a larger proportion of female patients had persistent symptoms at 3^24,27^ and 6 months.^20^ Education is also a demographic characteristic mentioned by some authors. Indeed, two studies report that low education is a factor of poorer prognosis at 12^14^ and 6 months.^20^ However, it is important to note that these two factors, female gender and education, are challenged by the study of Losoi et al. Rather, their results indicate that at 12 months, there was no difference in age, education, or gender between patients with mild PCS and those without symptoms.^21^

Older age is another factor on which there is disagreement between authors. As mentioned, Losoi et al. determined that age is not a significant factor in poor prognosis at 12 months.^21^ In contrast, three other studies reported the opposite result.^20,24,27^

History of psychiatric problems prior to the mTBI was also identified. Patients with preaccident psychiatric problems were more likely to complain of PCS, at 3^27^ and 6 months.^20^ The incidence of stressful events in a person’s life, as assessed by the *Stressful Life Events questionnaire,* was also a predictor of scores on the BSI depression and anxiety subscales at 3 months.^25^

Blood biomarkers were part of the initial assessment in three studies. One considered the presence of NSE and S-100B markers in serum within 6 h of mTBI,^17^ while another investigated the presence of blood-derived neurotrophic factors (BDNFs) within the first 24 h.^26^ Both conclude that blood biomarker levels influence the 6-month prognosis, i.e., elevated NSE and S-100B or low BDNF levels are predictive of poorer recovery. The third article analyzed 420 proteins in blood samples and concluded that 31, 43, 15, and 26 proteins were significantly associated with the poor recovery of neuropsychological symptoms at 6 months when measured at <72 h, 1 week, 1 month, and 3 months after the injury, respectively.^22^ However, no unique and common protein biomarker was identified. Finally, functional magnetic resonance imaging (fMRI) was a baseline measure used by two articles, which both determined that decreased connectivity between various and nonconsistent brain regions on fMRI is a predisposing factor for poorer recovery at 3^28^ and 12 months.^14^

## Discussion

This systematic review shows that symptom resolution has a heterogeneous prevalence according to the different studies, but the total proportion of recovered patients varies little between 1 and 6 months. Prognostic factors for poor recovery were identified, but some are not universally accepted.

The intensity and number of symptoms following an mTBI and the individual’s history of psychiatric problems are the most significant predictors of delayed recovery reported by the largest number of mostly moderate-quality studies.^14,17,19,20,24,27^ There is no clear consensus on the impact of gender, low education, and advanced age on recovery. The conclusions of the four moderate-risk articles citing these factors (three articles report female gender,^20,24,27^ three report age,^20,24,27^ and two report education^14,20^) are challenged by one article of low risk of bias.^21^ However, this study has only 75 participants with mTBI, which is not enough to contradict the set of four articles which total a much larger number of participants (*n* = 580 mTBI). It can therefore be assumed that age, female gender, and education probably have an impact on the prognosis of symptom recovery, but the literature is not yet sufficient on this subject. Remarkably, most included studies did not examine symptom severity as prognostic factor, either because they did not measure symptoms at baseline or because their primary objectives focused on other predictors. This suggests that symptom severity and intensity might have emerged as significant prognostic factors in more than just four studies had they been assessed more consistently. Additionally, all four studies linking symptom severity to prognosis measured it within the first week, suggesting timing may influence its predictive value. In contrast, one study measuring symptom severity within 72 h postinjury did not find it to be a prognostic factor, further highlighting the need for future research on the influence of early symptom assessment on recovery predictions. Regarding biomarkers and fMRI, studies seem to indicate that they are significant factors, but there were not enough high-quality studies to draw a strong conclusion.^14,17,22,26,28^

The prevalence of complete symptom resolution in patients is surprising. Indeed, as shown in Table 3, homogeneity in the total proportion of asymptomatic individuals can be observed. In other words, there is no recovery time that stands out where most participants had a full recovery.

At 1 month, the risk of bias of the total proportion can be considered moderate since two out of four have a moderate risk and the other two are opposites, i.e., low and high.^15,21,22,29^ Thus, because the proportion is based only on four articles totaling a relatively small population of 455 participants, our confidence level for the 1-month prognosis is rather low.

Subsequently, at 3 months, the risk of bias of the calculated proportion was judged to be low to moderate. Even though the article with the largest number of participants has a low risk of bias, two are of high risk, and the other are of moderate risk of bias.^15,22–24,28,29^ However, the total population of 665 participants across six articles allows for a possibly more robust conclusion. That said, our level of certainty about the strength of the results is moderate.

At 6 months, since the majority of articles have a moderate risk of bias and the only article with a low risk of bias has the smallest population among the six articles, the risk of bias of this grouping of results is considered moderate.^15,17,20–22,25,26^ With its 774 participants, this group has the largest total population among the four follow-up times, which increases the strength of its results. Despite this, our level of confidence is moderate given the quality of the articles included in this grouping.

Only two articles looked at the 12-month follow-up. This group is at moderate risk of bias because the study with the majority of the 165 participants (attrition rate of 0%^14^ and 20%^21^) in this grouping is at moderate risk of bias.^14,21^ Our level of confidence in this grouping is low due to the few studies and small sample that limits generalization to a larger mTBI population.

Several hypotheses can explain the similarity in the total proportions reported at 1, 3, 6, and 12 months. First, the large range in the proportions of recovered participants reported at each follow-up is one possible explanation (Table 3). For example, at 6 months, the study with the highest proportion of asymptomatic patients reports almost double the proportion of the lowest rate. However, this variability is not visible in the sum of recovered participants in this grouping. In addition, all articles included in this systematic review do not use the same methodology. The total proportion of recovered patients at each time point was obtained using data from different studies. Additionally, there may be differences in what each study considers as “asymptomatic,” given that they do not all use the same tools. For example, what is considered a “recovered” patient when measured with the SSS is not necessarily the same as with the RPQ. Differences in study designs may therefore have contributed to the similarity of total proportions.

Another hypothesis to explain these results could be an attrition bias due to a high lost to follow-up of recovered individuals.^32^ Indeed, it is possible that when participants no longer had symptoms, they stopped to participate in the study and did not return to study follow-ups. In addition, as mentioned above, study attrition is one of the domains most consistently rated as high risk of bias of the included studies. Therefore, it is possible that there were lost data, which had a direct impact on the results. Indeed, a high attrition phenomenon could explain why the prevalence of recovered patients seems to stagnate between 1 and 6 months, and only slightly increase at 12 months.

### Comparison with the literature

Several other studies have commented on the prognosis of symptom recovery following mTBI. A study by Cancelliere et al. showed that the presence of symptoms between 3 and 6 months using the RPQ or similar tools ranged from 18% to 31%.^32^ Symptom prevalence values vary depending on the use of a strict PCS definition (moderate PCS; 18%) compared with a broader definition (mild PCS; 31%). This represents a proportion of asymptomatic individuals that varies between 82% and 69%. These results are not consistent with those of the current study, which found a much lower proportion of asymptomatic patients at 3 and 6 months.

The differences in results can be explained by several reasons. Differences in methodology, attrition, mechanism and severity of trauma, and eligibility criteria can be some examples. Indeed, this systematic review^32^ used a population of adults presenting to emergency departments or trauma centers but does not specify what the exact population is, i.e., whether it is a nonathletic population. A large population of 11,742 participants across 43 studies were evaluated, which allows for greater generalization of the results.

The systematic review of the *Amsterdam International Consensus Statement on Concussion in Sports 2023,* which only included sport-related mTBI, found the same predictors of delayed recovery as the current study, namely the intensity and number of initial symptoms as the strongest predictors.^12^ Also, in agreement with this review, age and gender do not appear to be a significant factor in the prolonged recovery of individuals. It therefore seems that the prognostic factors predicting poorer recovery are similar between the athletic and nonathletic populations. However, the consensus’ conclusions on the prevalence of asymptomatic participants highly differ from those of this systematic review. Indeed, they suggest that symptom recovery is 14.0 days,^12^ highlighting the large difference in recovery between the two populations.

For studies conducted with an athletic population, other reasons may explain the differences in results. It can be hypothesized that sports teams with protocols post-mTBI would offer better management to athletes and thus a better recovery time, especially when they are supported by multidisciplinary teams to ensure return to play. The articles included in our literature review did not discuss protocols or interventions to optimize recovery time in the nonathletic population with mTBI. Thus, this may explain the difference in recovery time expressed by the consensus and that determined by this systematic review. It is also possible that younger healthy and fit sports individuals, with an active lifestyle and minimal number of comorbid health problems, were quicker to recover than the nonsport adult with a higher likelihood of comorbidities.

### Clinical relevance of findings

The identification of the proportion of asymptomatic patients during the various post-mTBI follow-ups also provides interesting insights. Indeed, although the literature commonly reports that normal recovery is <3 months,^5^ our findings suggest that about half of patients may have symptoms that persist for up to 6 months and up to 12 months for about 40% of them. These data can be used by clinicians to reassure patients that recovery extending to more than 3 months is a possible reality. Indeed, the presence of persistent symptoms following mTBI can be anxiety provoking in nearly 30% of patients, and therefore it may be relevant to present them with this type of statistic to explain that recovery varies greatly from one person to another.^33^

In addition, by highlighting the differences in the recovery of an athletic patient to that of a nonathlete patient, it is possible to see that the prognosis is not the same. Therefore, clinicians need to be able to manage patient expectations accordingly, by adjusting their message to the type of patient they are treating.

Prognosis after mTBI is important to help clinicians identify individuals at risk of a delayed recovery and adapt their management to optimize their recovery. Therefore, the identification of factors of delayed recovery such as the intensity and high number of initial symptoms and the presence of a psychiatric history should be considered by clinicians particularly during the initial questionnaire.

### Limitations

First, an important limitation of this study is that no recognized method has been used to determine the levels of evidence for our findings. Indeed, the use of a method such as the Grading of Recommendations Assessment, Development and Evaluation (GRADE) method could have supported the evidence found in relation to prognostic factors predicting poorer recovery after mTBI. The GRADE approach is used to define the quality of the evidence in terms of the certainty of the estimation of the effects of the prognostic factor(s) in question.^34^ As this step was not performed in this systematic review, it is not possible to determine the certainty of the evidence regarding the risk posed by the presence of a prognostic factor on recovery from mTBI.

Subsequently, the choice to use only PCS as a measure of recovery is also a limitation of this study. The presence of persistent symptoms following a concussion is certainly an important issue to consider, but recovery from mTBI can also be considered in a broader sense. Indeed, several studies use a different recovery measure and therefore had to be eliminated from this study. This is the case for several studies in the *Transforming Research and Clinical Knowledge in Traumatic Brain Injury* cohort, a longitudinal study between 2014 and 2018 aimed at demystifying the diagnosis and prognosis following a concussion. These studies are of high methodological quality and often include large cohorts of patients; as a result, their results are often of good quality. For example, three studies had to be excluded as they use the *Extended Glasgow Outcome Scale* as an outcome measure, and this tool does not measure symptoms but rather mTBI-related disabilities.^35–37^ It might have been relevant to include both disabilities and PCS to have a more holistic view of recovery after mTBI.

In addition, as previously noted, a common weakness of the included articles is participant attrition, which was often assessed with a high risk of bias; this raises doubts about the quality of our results. It is possible that some patients may not have completed their follow-up if they considered themselves recovered. This would strongly bias the results, particularly on the proportions of patients recovered at the various follow-ups established in our study. In fact, the overall risk of bias of studies could also be a possible source of error. To establish the proportion of recovered patients, only 11 of the 16 included studies had data that could be used, with most being of moderate risk of bias. Thus, it is a significant possibility that some biases have crept in and skewed the results. Finally, the heterogeneity of included studies, particularly in terms of methodologies, sample sizes, and outcome measures, makes direct comparisons challenging and prevents the performance of a meta-analysis. The discrepancies observed in predictors such as female gender, older age, and lower education may stem from these methodological differences, including variations in sample size and study design.

### Suggestions for future

There are a few possible solutions to improve scientific research in this field. First, it would be interesting to use a clear and consistent definition of recovery. Indeed, as reported in the Amsterdam Consensus, the wide variety of definitions of recovery in the different articles is a significant limitation to the conclusions drawn.^10^ Standardizing the definition of recovery would allow for more accurate and unanimous conclusions related to recovery time and prognostic factors. In addition, the use of the same follow-up points over time, for example to always assess the number of people recovered at 1, 3, and 6 months, could make it possible to distinguish exactly the precise time of recovery of individuals since the studies would be easily comparable with each other. Subsequently, it would be relevant for more high-quality longitudinal and prospective studies to be carried out, to minimize the risk of bias and thus optimize the quality of the results. In addition, the sample size should be large enough and, most importantly, have low attrition to ensure the applicability of their findings to the target population.

## Conclusion

This study concludes that the proportions of patients with complete resolution of symptoms after mTBI in the nonathletic population range from 55.6% to 61.8% between 1 and 12 months and reach a minimum proportion of 52.3% at 6 months. Thus, this suggests that about half of patients will become asymptomatic in the first 6 months following mTBI. The most significant prognostic factors in predicting poorer recovery in this population are the intensity and number of symptoms as well as psychological history. Further studies are needed to determine with certainty the impact of other prognostic factors reported in the literature. Individualized patient care should be prioritized to maximize the recovery of individuals with mTBI who have risk factors for prolonged recovery.

## Transparency, Rigor, and Reproducibility

This study demonstrates a strong commitment to transparency by thoroughly documenting the methodology used for the systematic review. The search strategy, which involved the Embase, OVID Medline, and CINHAL databases, is clearly described, along with the specific search strategy employed (Supplementary Table S1). The inclusion and exclusion criteria, as well as the step-by-step screening process using Covidence software, are explicitly detailed to ensure that the selection of studies is reproducible. Additionally, the risk of bias for each included study was systematically evaluated using the QUIPS Risk of Bias Assessment Tool, with domain-specific and overall ratings provided in Table 2. Comprehensive data tables and supplementary materials further enhance transparency by allowing other researchers to scrutinize the underlying evidence and decisions made throughout the review.

The rigor of this review is evident in the adherence to established guidelines for systematic reviews and the use of validated tools to assess recovery outcomes and prognostic factors. Data extraction focused on standardized outcome measures, such as the Rivermead Post-Concussion Questionnaire and the Brief Symptom Inventory, ensuring the reliability of the findings. Covidence software was employed to manage the screening and selection phases, reducing variability, and improving the consistency of study inclusion decisions. The assessment of risk of bias across six domains using a validated framework ensured that methodological limitations were carefully considered in the synthesis of results. This methodological rigor underpins the validity and reliability of the study’s conclusions about recovery trajectories and prognostic factors following mTBI.

To facilitate reproducibility, the study provides detailed descriptions of the search strategy, inclusion/exclusion criteria, and data extraction processes, enabling other researchers to replicate the review. A consistent definition of recovery, aligned with established literature, was used to standardize outcome assessments across included studies. The analytical methods used to calculate pooled recovery proportions and identify significant prognostic factors are transparently reported, with results presented in tables and supplementary materials. By offering clear documentation and access to key data, this study ensures that its findings can be independently verified and utilized as a foundation for further research in mTBI recovery and prognosis.

## Abbreviations Used

BDI: Beck Depression Inventory
BDNFs: Blood-Derived Neurotrophic Factors
BSI: Brief Symptom Inventory
fMRI: functional Magnetic Resonance Imaging
GCS: Glasgow Coma Scale
GRADE: Grading of Recommendations Assessment, Development and Evaluation
GSI: Global Severity Index
mTBI: mild Traumatic Brain Injury
PCS: Post-Concussion symptoms
PTSD: Post-Traumatic Stress Disorders
QUIPS: QUality In Prognostic Studies
RPQ: Rivermead Postconcussion Questionnaire
SSS: Symptom Severity Score
SCAT-2: Sport Concussion Assessment Tool-2
SD: Standard Deviation
VAS: Visual Analogue Scale
