# Supplementary material for: Recovery from Mild Traumatic Brain Injury in the Nonathletic Population: A Systematic Review
Source: Neurotrauma Rep. 2025 Apr 24;6(1):355–74. doi: 10.1089/neur.2025.0006 (PMC12281108; doi:10.1089/neur.2025.0006)
Supplement: Supplementary Table S1 [file neur.2025.0006_supplementary_table_s1.docx]

**Supplementary Table 1: Search Strategy**

| **Database** |  |  | **Keywords** | **Number of results** |
| --- | --- | --- | --- | --- |
| EMBASE | 1st concept | 1 | "mild traumatic brain injur*" OR "mTBI" OR "concussion*" OR "brain commotion*" OR "cerebral commotion*" OR "post?concuss* symptom*" OR "post?concuss* disorder*" OR "post?concuss* syndrome" | 20 938 |
|  |  | 2 | ('brain concussion'/exp OR 'postconcussion syndrome'/exp) | 10 179 |
|  |  | 3 | 1 OR 2 | 23 875 |
|  | 2nd concept | 4 | Prognos* OR Prediction* | 1 621 760 |
|  |  | 5 | prognosis'/exp | 895 365 |
|  |  | 6 | 4 OR 5 | 1 862 731 |
|  | 3rd concept | 7 | Recover* OR rehabilitat* OR remission* OR convalesc* | 1 511 004 |
|  |  | 8 | convalescence'/exp | 56 944 |
|  |  | 9 | 7 OR 8 | 1 526 608 |
|  | Final search | | 3 AND 6 AND 9 | 603 |
| MEDLINE | 1st concept | 1 | ("mild traumatic brain injur*" OR "mTBI" OR "concussion*" OR "brain commotion*" OR "cerebral commotion*" OR "post?concuss* symptom*" OR "post?concuss* disorder*" OR "post?concuss* syndrome").ti,ab | 14 809 |
|  |  | 2 | "Brain concussion"/ OR "post-concussion syndrome"/ | 11 802 |
|  |  | 3 | 1 OR 2 | 17 931 |
|  | 2^nd^ concept | 4 | (Prognos* OR Prediction*).ti,ab | 1 136 072 |
|  |  | 5 | "Prognosis"/ | 585 614 |
|  |  | 6 | 4 OR 5 | 1 408 580 |
|  | 3rd concept | 7 | (Recover* OR rehabilitat* OR remission* OR convalesc*).ti,ab | 1 094 201 |
|  |  | 8 | "Recovery of function"/ | 58 858 |
|  |  | 9 | 7 OR 8 | 1 121 904 |
|  | Final search | | 3 AND 6 AND 9 | 423 |
| CINHAL | 1st concept | 1 | "mild traumatic brain injur*" OR "mTBI" OR "concussion*" OR "brain commotion*" OR "cerebral commotion*" OR "post?concuss* symptom*" OR "post?concuss* disorder*" OR "post?concuss* syndrome" | 8 370 |
|  |  | 2 | (MH "Brain Concussion") OR (MH "Postconcussion Syndrome") | 6 616 |
|  |  | 3 | 1 OR 2 | 9 617 |
|  | 2nd concept | 4 | Prognos* OR Prediction* | 201 525 |
|  |  | 5 | (MH "Prognosis") | 95 622 |
|  |  | 6 | 4 OR 5 | 253 209 |
|  | 3rd concept | 7 | Recover* OR rehabilitat* OR remission* OR convalesc* | 245 741 |
|  |  | 8 | (MH "Recovery") | 38 417 |
|  |  | 9 | 7 OR 8 | 261 145 |
|  | Final search | | 3 AND 6 AND 9 | 136 |
